# Comfrey root: from tradition to modern clinical trials

**DOI:** 10.1007/s10354-012-0162-4

**Published:** 2012-12-07

**Authors:** Christiane Staiger

**Affiliations:** Merck Selbstmedikation GmbH, Rößlerstr. 96, 64293 Darmstadt, Germany

**Keywords:** Review, *Symphytum officinale* L., Comfrey root, Clinical trial, Non-interventional study, Efficacy, Safety, Übersicht, *Symphytum officinale* L., Beinwellwurzel, Klinische Studie, Nicht-interventionelle Studie, Wirksamkeit, Sicherheit

## Abstract

Comfrey (*Symphytum officinale* L.) has been used over many centuries as a medicinal plant. In particular, the use of the root has a longstanding tradition. Today, several randomised controlled trials have demonstrated the efficacy and safety. Comfrey root extract has been used for the topical treatment of painful muscle and joint complaints. It is clinically proven to relieve pain, inflammation and swelling of muscles and joints in the case of degenerative arthritis, acute myalgia in the back, sprains, contusions and strains after sports injuries and accidents, also in children aged 3 years and older. This paper provides information on clinical trials, non-interventional studies and further literature published on comfrey root till date.

## Abstract (de)

Die arzneiliche Verwendung von Beinwellwurzel hat eine jahrhundertealte Tradition. Heute belegen zahlreiche randomisierte klinische Prüfungen die Wirksamkeit und Sicherheit. Beinwellwurzelextrakt wird äußerlich angewendet zur Behandlung von schmerzhaften Muskel- und Gelenkbeschwerden. Schmerzen, Entzündungen und Schwellungen bei Kniegelenksarthrose degenerativen Ursprungs und akute Myalgien im Bereich des Rückens gehören ebenso zu den klinisch belegten Anwendungsgebieten wie Verstauchungen, Prellungen und Zerrungen nach Sport- und Unfallverletzungen, auch bei Kindern ab 3 Jahren. Der Artikel gibt eine aktuelle Übersicht über klinische Prüfungen, nicht-interventionelle Studien und weitere Literatur zu Beinwellwurzel.

## Introduction

Comfrey root has been used as a traditional medicinal plant for the treatment of painful muscle and joint complaints for centuries [1, 2]. Native in Europe, the plant has an impressive record of medicinal use. It also naturalised in Northern America, where it rapidly spread. Native Americans recognised the healing powers and included comfrey root in their therapeutic armamentarium [3–5]. A recent text book chapter gives detailed information on botanical aspects and harvesting of the plant [6].

The key activity-determining constituents of comfrey root extracts and its molecular mechanisms of action have not been completely elucidated. Allantoin and rosmarinic acid are probably of central importance for its pharmacodynamic effects [7]. The German Commission E [8] has assessed Symphyti radix (comfrey root) deriving from *Symphytum officinale* L. positively for the external use in bruises, strains and sprains and acknowledged its actions as anti-inflammatory, antimitotic and promotion of callus formation.

Further, a European Scientific Cooperative on Phytotherapy Monograph (ESCOP) is available for comfrey root [9]. The monograph mentions strains, contusions and distortions, osteoarthritis (OA), epicondylitis, tendovaginitis and periarthritis as therapeutic indications substantiated by clinical trials. ESCOP states that comfrey root has also been used for tendinitis syndrome, knee joint injuries, non-active gonarthrosis, insect bites, mastitis, fractures and skin inflammation, although published scientific evidence does not yet adequately support these indications.

## Randomised clinical trials

The medicinal use of preparations from the underground parts of the plants (Symphyti radix) is well established. Till date, comfrey root extract preparations have been marketed in more than ten countries. Most recently, a cream has also been launched in Austria. Randomised clinical trials and non-interventional studies studied the efficacy of comfrey root extract ointment for the treatment of various muscle and joint complaints [10].

### Back pain

Back pain, especially of the upper and lower back, is a widespread condition impairing quality of life and functional movement in a large number of individuals. The treatment strategy has recently also adopted a direct anti-inflammatory topical approach, mostly with chemical non-steroidal anti-inflammatory drugs (NSAIDs). This approach has been intensively used and has proved to be efficacious in the management of symptoms, thus reducing pain, facilitating rehabilitation and achieving earlier recovery. Comfrey root as a herbal ingredient can contribute in the same way, as it is also known for its anti-inflammatory properties.

A double-blind, placebo-controlled, multi-centre, randomised clinical trial with parallel group design was conducted over a period of 5 days [11]. Total 120 patients with acute upper or lower back pain used either a verum cream containing comfrey root fluid extract (1:2, 35.0 g, extraction solvent ethanol 60 % (v/v), less than 0.35 ppm of pyrrolizidine alkaloids) or a corresponding placebo. They were treated three times a day, 4 g per application. The trial included four visits and was performed at the Deutsche Sporthochschule in Cologne and three additional ambulatory centres for orthopaedics and sports medicine.

The primary efficacy variable was the area under the curve (AUC) of the visual analogue scale (VAS) on active standardised movement values at first to fourth visit. Patients performed standardised, muscle group-specific tests to assess the pain intensity on VAS. Secondary objectives were back pain at rest, pressure algometry, global assessment of efficacy by the patient and the investigator, intake of analgesic medication and functional impairment measured using the Oswestry Disability Index.

The results showed a significant treatment difference between comfrey root extract and placebo regarding the primary variables. The pain intensity on active standardised movement decreased on average (median) approximately 95.2 % in the comfrey extract group (104.8–12.7 mm (mean VAS sum)) and 37.8 % in the placebo group (100.0–56.5 mm (mean VAS sum); *p* < 0.001). Also, in all secondary parameters, the superiority of the verum treatment compared with placebo was significant (each *p* < 0.001). Both the AUC of the reported back pain at rest, the AUC of the pressure algometry in the trigger point as well as the global assessment of the efficacy by the patients and the investigators showed a clinically relevant effect in reducing acute back pain. For the first time, also a fast-acting effect of the ointment (1 h) was witnessed. After 1 h the pain intensity was already decreased about 33.0 % in the comfrey group (104.8–60.4 mm (mean VAS sum)) and 12.0 % in the placebo group (100.00–86.5 (mean VAS sum)) indicating an early onset of the treatment effect. Four patients in the comfrey extract group and three in the placebo group experienced adverse events in the course of the clinical trial. Eczema, cold, nausea and rhinitis occurred in the verum group, headache (*n* = 2) and pruritus in the placebo group. All adverse events were of mild severity. One comment on the trial asked for more data in patients with different sorts of other back pain, but admits that the results are relevant and topical treatment is increasingly considered as a serious treatment option [12].

### Osteoarthritis (OA)

The same comfrey root extract cream was investigated in a randomised, double-blind trial including 220 patients suffering from painful OA of the knee [13]. OA characterises a primarily non-inflammatory, degenerative change of the structure of the cartilage and bones of one or more joints, involving an increasing deformation of the joint. On principle, all joints can be affected; most frequently knee and hip joints, hands and the spine. As age is one of the strongest risk factors for OA of all joints, the frequency of OA rises with age [14]. Approximately, 80 % of people more than 75 are affected. Symptomatic improvement represents an important therapeutic objective. The reduction of pain, the preservation and the restoration of the joint’s function and thus the restoration of quality of life are therefore important target parameters. Again, topical treatment is one further option besides oral medication.

All patients in this clinical trial met the criteria of the American College of Rheumatology. They received 2 g of either the active or a corresponding placebo cream three times daily, for 21 days. In self-medication, pain, functional impairment and stiffness are the most important symptoms patients seek to relieve. Therefore, the primary target variable was the VAS sum score of pain at rest and pain on movement. Secondary target variable was the Western Ontario and McMaster Universities Osteoarthritis Index (WOMAC) score. The total score of the primary target variable decreased by 51.6 mm (54.7 %) in the verum group and 10.1 mm (10.7 %) in the placebo group, a significant difference of 41.5 mm (44.0 %) between groups (*p* < 0.001). The secondary target criterion reduced by 60.4 mm (58.0 %) in the verum group and 14.7 mm (14.1 %) in the placebo group during the course of the study, the difference of 45.7 mm (43.9 %) being again significant (*p* < 0.001). Superiority of improvement in the verum group was also evident with respect to four explorative secondary parameters: SF-36 (quality of life), angle measurement (mobility of the knee), clinical global impression (CGI) and global assessment of efficacy by physicians and patients (*p* < 0.001 for each parameter). A total of 22 adverse events (AE) occurred in 22 patients (7 in the active therapy group, 15 in the placebo group). None did represent an adverse drug reaction in the active therapy group.

Usually, the production of placebos for herbal drugs is associated with difficulties. However, one comment on the trial emphasized that due to the low inherent smell of the extract and the same perfume used in both placebo and verum, a very good blinding could be achieved for this preparation [15]. Another comment found the trial to be well conducted and in accordance with the GCP-ICH-guidelines and sees the short-term use of the preparation to be a useful treatment option free of serious adverse reactions [16].

Another recent trial investigated two concentrations of topical, comfrey-based botanical creams containing a blend of tannic acid and eucalyptus to a eucalyptus reference cream on pain, stiffness and physical functioning in those with primary OA of the knee [17]. Total 43 male and female subjects (45–83 years old) with diagnosed primary OA of the knee who met the inclusion criteria were randomly assigned to treatment groups: 10 or 20 % comfrey root extract (*Symphytum officinale* L.) or a placebo cream. Outcomes of pain, stiffness and functioning were measured on the WOMAC. Participants applied the cream three times a day for 6 weeks and were evaluated every 2 weeks during the treatment. Repeated-measures analyses of variance yielded significant differences in all of the WOMAC categories (pain *p* < 0.01, stiffness *p* < 0.01 and daily function *p* < 0.01), confirming that the 10 and 20 % comfrey-based creams were superior to the reference cream. The active groups each had two participants who had temporary and minor adverse reactions of skin rash and itching, which were rapidly resolved by modifying applications. Authors concluded that both active topical comfrey formulations were effective in relieving pain and stiffness and in improving physical functioning and were superior to placebo in those with primary OA of the knee without serious adverse effects.

### Blunt injuries

A further trial with a combination of standardized comfrey extract (200 mg/g), tannic acid (100 mg/g) plus other ingredients including aloe vera gel (300 mg/g), eucalyptus oil (40 mg/g), and frankincense oil (1.0 mg/g) assessed the efficacy of thrice daily topical treatment on osteoarthritic knee pain, markers of inflammation and cartilage breakdown over 12 weeks [18]. Adults aged 50–80 years (*n* = 133) with clinical knee OA received verum or placebo in addition to existing medications. Pain and function were measured using a VAS and the Knee Injury and Osteoarthritis Outcome Score (KOOS) scale at baseline, 4, 8 and 12 weeks. Inflammation was measured analysing IL-6 expression and CTX-2 presence as representative for cartilage breakdown using ELISA, at baseline and 12 weeks. Although the paper does not specify the part of the comfrey plant used in the extract, the drug extract ratio or the nature of standardization, it refers to the same commercial product as Smith and Jacobson [17]. Pain scores were significantly lower in the verum group compared to placebo after 12 weeks using both the VAS (−9.9 mm, *p* = 0.034) and the KOOS pain scale (+5.7, *p* = 0.047). Changes in IL-6 and CTX-2 were not significant (−0.04, P = 0.5; −0.01, *p* = 0.68). In a post-hoc analysis, the authors suggested that treatment may be most effective in women (VAS −16.8 mm, *p* = 0.008) and those with milder radiographic OA (VAS −16.1 mm, *p* = 0.009). They concluded that the topical comfrey combination with tannic acid is a safe and effective treatment for the symptoms of knee OA in participants with moderate knee pain, and clinical OA. The treatment reduced pain and increased muscle strength, but had no effect on systemic inflammation or cartilage breakdown over 12 weeks of treatment.

Comfrey root has a strong historical record in the treatment of blunt injuries due to its anti-inflammatory, de-swelling and pain-relieving properties. The efficacy granted by the Commission E has been further substantiated with clinical data. The percutaneous efficacy of the cream with the afore-mentioned comfrey root fluid extract (1:2, 35.0 g, extraction solvent ethanol 60 % (v/v)) was confirmed in a clinical trial on patients suffering from ankle distortion [19, 20]. The double-blind, multi-centre, randomised, placebo-controlled, group comparison included 142 patients with a mean age of 31.8 years; among them 78.9 % were male. The inclusion criterion was an uncomplicated, acute unilateral ankle distortion that had been endured no longer than 6 h previously. The duration of treatment was 8 days. The afflicted ankle was topically treated with c. 2 g (corresponding to a 6 cm strand of cream) of either verum or placebo.

The primary variable, tenderness of the ankle joint, was measured by pressure algometry, meaning the difference in tolerated pressure between injured and healthy ankles. Under active treatment, no adverse drug reactions were reported. During the course of treatment, pain regressed significantly more in the comfrey extract group than in the placebo group (*p* < 0.0001) and at the final assessment the reductions in tenderness compared with initial values were 2.44 kp/cm^2^ in the verum group compared with only 0.95 kp/cm^2^ in the placebo group. Compared with placebo, superiority of the verum treatment was significant with regard to reduction in pressure pain (tonometric method, *p* < 0.0001), ankle oedema (figure-of-eight method, *p* = 0.0001), ankle mobility (dorsiflexion, *p* = 0.002; plantar flexion, *p* = 0.0116) and global efficacy (*p* < 0.0001).

### Verum-controlled versus diclofenac

For comfrey root, also a verum-controlled clinical trial versus topical diclofenac was performed. In a single-blind, controlled, randomised, parallel groups, multi-centre and confirmatory clinical trial outpatients with acute unilateral ankle sprains (*n* = 164) received either a 6 cm-long ointment layer of the above-mentioned comfrey root extract cream (*n* = 82) or of diclofenac gel containing 1.16 g of diclofenac diethylamine salt (*n* = 82) [21]. The patients applied the cream four times a day for 7 days.

The primary efficacy variable was pain arising from pressure on the injured area, measured with a calibrated algometer on days 0, 4 and 7 and evaluated by the AUC of the pain-time curve. Secondary variables were the circumference of the joint (swelling, figure-of-eight method), the individual spontaneous pain sensation at rest and at movement according to a VAS, the global efficacy evaluation, the global assessment of tolerability and further variables. It was confirmatorily shown that comfrey extract is non-inferior to diclofenac.

The 95 % confidence interval for the AUC (comfrey extract minus diclofenac gel) was 19.08–103.09 h*N/cm^2^ and completely above the margin of non-inferiority. After 7 days of treatment, a mean relative reduction in VAS at rest of 92 % was found in the comfrey cream group. The corresponding reduction in the diclofenac group was 85 %. The mean relative reductions in VAS in motion were 83.2 % for comfrey extract and 72.4 % for diclofenac. The ankle swelling was decreased by 79.5 % in the comfrey root and 69.4 % in the diclofenac group. The pain on pressure measured with an algometer was reduced by 80.6 % in the comfrey root, but only by 74.7 % in the diclofenac group.

A re-evaluation of the trial data in accordance with CPMP-guidelines [22] revealed even a superiority of the herbal in several parameters [23]. In the primary variable, the comfrey root extract cream showed a statistically significant superiority above the diclofenac gel (*p* = 0.0012). On day 4, a statistically significant reduction of the pain on pressure (*p* = 0.0449), and on day 4 (*p* = 0.0368) and day 7 (*p* = 0.0074) a statistically significant reduction of the pain on movement was recorded. Further, the physicians (*p* = 0.0130) as well as the patients (*p* = 0.0111) rated the global efficacy of the comfrey preparation significantly higher than the efficacy of the diclofenac gel.

Diclofenac and other NSAIDs are well known for their side effects and upper gastrointestinal (GI) toxicity. There is also documented data regarding their adverse effects on lower GI tract such as colonic strictures, inflammatory bowel disease and complications of diverticular disease in the form of abscess or perforation. Therefore, patients and physicians seek for therapeutic alternatives. The findings of this clinical trial substantiates comfrey root extract as a good and efficient option for alternative topical treatment.

### Combination with methyl nicotinate

A topical combination of 35 % comfrey root extract plus 1.2 % methyl nicotinate was compared versus a single preparation of methyl nicotinate or placebo cream for relief of acute upper or lower back pain [24] in a randomised, multi-centre, double-blind, three-arm, placebo-controlled trial. Total 379 patients were randomly assigned to three groups (combination, *n* = 163; methyl nicotinate, *n* = 164 and placebo, *n* = 52). They applied a 12 cm layer of cream three times daily for 5 days. The primary efficacy variable was the AUC of the VAS on active standardised movement values at first to fourth visit. Secondary measures included back pain at rest, pressure algometry, consumption of analgesic medication, functional impairment measured with Oswestry Disability Index and global assessment of response.

The AUC of the VAS on active standardised movement was markedly smaller in the combination treatment group than in the methyl nicotinate and in the placebo group (ANOVA: *p* < 0.0001). The pair-wise comparisons of the mean AUCs of VAS sums on active standardised movement showed values 27 % lower in favour of the combination compared with methyl nicotinate (6548.65 mm × h versus 8975.32 mm × h, i.e. a mean treatment effect of - 2426.7 mm × h), and values 50 % lower in favour of the combination compared to placebo (6548.65 mm × h versus 13052.40 mm × h, mean treatment effect - 6503.8 mm × h). Methyl nicotinate alone reached a reduction in this variable of 31 % compared with placebo (8975.32 mm × h versus 13052.40 mm × h, mean treatment effect 4077.1 mm × h). All pair-wise comparisons were statistically significant (*t*-test: *p* < 0.0001).

**Figure 1:**
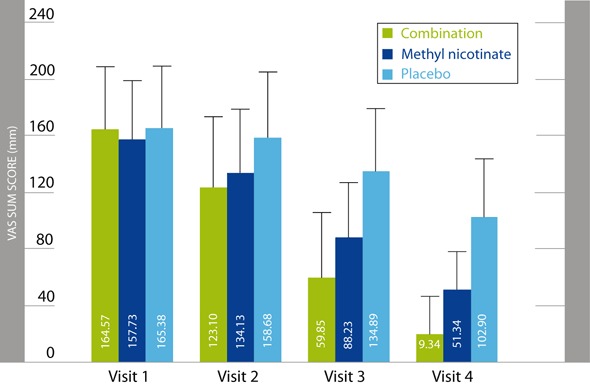

The VAS sum score pain on active movement decreased from visit 1 to visit 4 by 145.2 mm (88.2 %) in the combination group, 106.4 mm (67.5 %) in the methyl nicotinate group and 62.5 mm (37.8 %) in the placebo group (Fig. 1). Back pain at rest clearly decreased from visit 1 to visit 4 by 45.2 mm (91.5 %) in the combination group, 34.8 mm (74 %) in the methyl nicotinate group and 19.3 mm (39.2 %) in the placebo group (Fig. 1).

The combination demonstrated superiority to the two other treatment arms, while methyl nicotinate displayed a considerable effect as well.

### Other clinical trials

In an earlier 4-week pilot study, 41 patients with different forms of musculoskeletal rheumatism (mainly epicondylitis, tendovaginitis and periarthritis) were treated topically with the same cream as mentioned above (*n* = 20) or with placebo (*n* = 21) [25]. Efficacy was assessed using several pain parameters: tenderness when pressure applied, pain at rest and during exercise. With respect to “tenderness when pressure applied”, the ointment proved superior to placebo in patients with epicondylitis and tendovaginitis, but not in patients with periarthritis.

The effects of dermatological preparations containing 5 or 10 % of a comfrey root extract (2:7, 50 % ethanol) on the process of healing of experimentally induced UV-B erythema were studied in 29 volunteers in a controlled pharmacological trial. The anti-inflammatory potency of the extract was found to be equal to or greater than that of diclofenac. A positive correlation could be demonstrated between efficacy and the concentration of a-hydroxy caffeic acid in the extract, but not for allantoin [7, 26].

Comfrey root has also been used for knee joint injuries and non-active gonarthrosis, further in the treatment of tendinitis syndrome, insect bites, mastitis, fractures, skin inflammation, multiple abscesses of sweat glands, gangrenous ecthymas, furuncles, dicubital ulcers and chronic varicose ulceration, as prior studies and individual case reports reflect [10].

## Post-marketing surveillance

### Children

In a non-interventional study of a comfrey root extract cream containing 35 % of a comfrey root extract (1:2, ethanol 60 % (v/v)), the tolerability and efficacy were examined in 306 children aged 3–12 years [27]. The preparation was used for a variety of conditions such as contusions (61.4 %), strains (14.1 %), distortions (30.4 %) and other indications (6.9 %). Most children applied the ointment three times daily (57.8 %), few four times daily (26.1 %) or twice a day (13.4 %). Thereby the physicians administered mostly the same dosages as for adults and children of 12 years of age and older. In the overall score of the findings pain on palpation, restriction of movement and haematoma manifestation (minimum 3, maximum 15) a notable improvement in the clinical result became clear: The initial value of 10.61 fell by 6.18 points or by 58.3 %. Clear remission or improvement was revealed in every individual finding. For all clinical symptoms, an improvement of more than 50 % could be calculated. The most markedly reduction was in pain at rest (62.6 %), restriction of movement (62.0 %) and pain sensitivity (61.4 %).

### Comfrey cream

In a non-interventional post-marketing surveillance study, 163 patients with a mean age of 45.3 years applied the same comfrey root extract cream for several conditions, the most frequent being contusions (33.1 %), painful joint complaints (27.6 %), sprains (26.4 %) and painful muscle complaints (23.3 %) [28]. Most patients applied the preparation two (38 %) or three (48.5 %) times daily and the median duration of treatment was 11.5 days. During the observation period, symptoms of pain at rest and during the night, pain during motion, tenderness when pressure applied, impaired mobility, painful muscle complaints and swellings improved markedly. Morning stiffness of the joints decreased by 94 % from 17 min initially to 1 min. The use of NSAIDs was reduced or discontinued by 13.5 % of patients. The physicians assessed global efficacy as excellent in 38.7 % of cases and good in 54.6 %.

### Comfrey paste

In a simultaneous surveillance study, 162 patients applied a similar preparation, a paste containing 30 % of the above-mentioned fluid extract of comfrey roots [29]. They as well treated a variety of conditions such as painful joint complaints (34 %), contusions (26.5 %) or painful muscle complaints (21.6 %). Most patients applied the preparation once (23.5 %) or twice (52.5 %) daily and the median duration of treatment was 11.8 days. Again, symptoms of pain at rest and pain during movement, impaired mobility, swelling and painful muscle complaints improved markedly during the observation period. Morning stiffness of investigated joints decreased by 90 % from 20 min initially to 2 min. The use of NSAIDs was reduced or discontinued by 21 % of patients. Global efficacy was assessed by the physicians as excellent in 65.4 % of cases and good in 32.7 %.

### Combination with methyl nicotinate

A cream consisting of a combination of 35 % of comfrey root fluid extract and 1.2 % methyl nicotinate is available in Germany, Luxembourg and Switzerland. Another simultaneous non-interventional study included 162 patients with a mean age of 49.7 years [30]. The mean duration of treatment was 12.3 days. Pain at rest and during the night was reduced by 45 %, pain during motion by 47 %, tenderness when pressure applied by 47 %, painful muscle complaints by 48 % and impaired mobility improved by 46 %. In the course of the study, seven non-serious, resolved adverse events, namely skin reactions such as redness or itching, were recorded in four patients.

## Safety

Literature on comfrey often focuses on the content of pyrrolizidine alkaloids (PA) of the raw plant material. It is important to note that fully licensed medicinal products available today contain depleted or PA-free processed extracts. In fact, using these approved and safe products, pyrrolizidine alkaloids are no longer of clinical significance. Still, some authors recommend a restriction of the duration of treatment, also with externally applied comfrey preparations. In Germany, the restriction limiting application to 4–6 weeks/year applies only to preparations containing more than 10 mg, but less than 100 mg pyrrolizidine alkaloids (daily allowance). The application of modern preparations results in far below the daily allowance of 10 mg. As a consequence, there are no restrictions in Germany on these products as regards the duration of treatment [31].

The absence of genotoxic effects was demonstrated in the bacterial reverse mutation assay (Ames test) for the PA-free liquid extract used in the above-mentioned clinical trials and studies [32]. The extract was investigated for its ability to induce gene mutations in *Salmonella typhimurium* strains TA 98, TA 100, TA 102, TA 1535 and TA 1537 with and without metabolic activation using the mammalian microsomal fraction S9 mix. Reference mutagens were used to check the validity of the experiments. The comfrey root extract showed no biologically relevant increases in revertant colony numbers of any of the five tester strains, neither in the presence nor in the absence of metabolic activation. In conclusion, the fluid extract was not mutagenic in the bacterial reverse mutation assay.

## Conclusion

Today, modern clinical data substantiates the traditional topical application of comfrey root preparations. Several recent randomised clinical trials confirmed the efficacy in the treatment of pain, inflammation and swelling in the case of degenerative arthritis, acute myalgia in the back, sprains, contusions and strains after sports injuries and accidents, also in children aged 3 years and older. Comfrey root is a valuable and rational therapeutic option for patients suffering from muscles and joint pain [33]. Coming from a herbal, representing rational phytotherapy, but combining very good efficacy and safety it should be a part of physicians’ standard treatment tool box.

### Conflict of interest

Christiane Staiger is an employee of Merck Selbstmedikation GmbH, Darmstadt, Germany. The company has licensed medicines containing comfrey root extract in several countries.
